# Effect of movement‐evoked and tonic experimental pain on muscle force production

**DOI:** 10.1111/sms.14509

**Published:** 2023-10-06

**Authors:** Hélio V. Cabral, Valter Devecchi, Chelsea Oxendale, Ned Jenkinson, Deborah Falla, Alessio Gallina

**Affiliations:** ^1^ School of Sport, Exercise and Rehabilitation Sciences College of Life and Environmental Sciences, University of Birmingham Birmingham UK; ^2^ Centre of Precision Rehabilitation for Spinal Pain College of Life and Environmental Sciences, University of Birmingham Birmingham UK; ^3^ Department of Clinical and Experimental Sciences Università degli Studi di Brescia Brescia Italy; ^4^ Department of Sport and Exercise Sciences University of Chester Chester UK; ^5^ Centre for Human Brain Health, College of Life and Environmental Sciences University of Birmingham Birmingham UK

**Keywords:** electrical stimulation, knee, motor adaptation, pain, torque

## Abstract

**Introduction:**

When performing an exercise or a functional test, pain that is evoked by movement or muscle contraction could be a stronger stimulus for changing how individuals move compared to tonic pain. We investigated whether the decrease in muscle force production is larger when experimentally‐induced knee pain is directly associated to the torque produced (movement‐evoked) compared to a constant painful stimulation (tonic).

**Methods:**

Twenty‐one participants performed three isometric knee extension maximal voluntary contractions without pain (baseline), during pain, and after pain. Knee pain was induced using sinusoidal electrical stimuli at 10 Hz over the infrapatellar fat pad, applied continuously or modulated proportionally to the knee extension torque. Peak torque and contraction duration were averaged across repetitions and normalized to baseline.

**Results:**

During tonic pain, participants reported lower pain intensity during the contraction than at rest (*p* < 0.001), whereas pain intensity increased with contraction during movement‐evoked pain (*p* < 0.001). Knee extension torque decreased during both pain conditions (*p* < 0.001), but a larger reduction was observed during movement‐evoked compared to tonic pain (*p* < 0.001). Participants produced torque for longer during tonic compared to movement‐evoked pain (*p* = 0.005).

**Conclusion:**

Our results indicate that movement‐evoked pain was a more potent stimulus to reduce knee extension torque than tonic pain. The longer contraction time observed during tonic pain may be a result of a lower perceived pain intensity during muscle contraction. Overall, our results suggest different motor adaptation to tonic and movement‐evoked pain and support the notion that motor adaptation to pain is a purposeful strategy to limit pain. This mechanistic evidence suggests that individuals experiencing prevalently tonic or movement‐evoked pain may exhibit different motor adaptations, which may be important for exercise prescription.

## 1 INTRODUCTION

Knee pain is a common cause of decreased sport participation,^1^ absence from competition,^2^ and a key symptom to monitor recovery after sport injuries.^3^ Since painful stimuli are interpreted as potential threats to body integrity, individuals adopt a range of motor adaptations from movement avoidance to subtle changes in motor strategies, with the common goal of protecting the potentially injured tissue.^4^ The direct association between movement and pain as a potential driver for motor adaptation prompted several groups to propose a shift of focus from “pain and movement” to “pain with movement”.^5^, ^6^ This is specifically relevant during exercise and sport, where movement can cause an increase, a decrease, or be unrelated to pain. For instance, patellofemoral pain is diagnosed as pain that is exacerbated by load‐bearing activities,^7^ but a large proportion of individuals also report pain at rest.^8^ Similarly, individuals with patellar tendinopathy report sudden increases of pain when landing from a jump,^9^ but sustained isometric contractions alleviate the pain sensation over time.^10^ If motor adaptation occurs to limit pain,^4^ these heterogeneous pain presentations would necessarily lead to different motor adaptations. A better understanding of whether motor adaptation to pain depends on the association between movement and pain could help develop tailored exercise prescriptions in individuals with pain.

Experimental pain models have been widely used in healthy individuals to investigate how the central nervous system adapts to different types of pain. A consistent finding across systematic reviews is that most of the studies that investigated motor adaptation to pain have used experimental models of tonic pain.^11^, ^12^ These approaches have led to significant advances in our understanding of how the central nervous system adapts to a tonic nociceptive input. However, spontaneous and movement‐induced pain are defined as different features of musculoskeletal pain,^13^ and therefore tonic pain models may not accurately replicate the motor adaptations induced by movement‐evoked pain, which is commonly observed in pathologies such as patellofemoral pain^7^ and patellar tendinopathy.^14^ In fact, it has been reported that pain induced with injection of hypertonic saline solution is often alleviated by movement or muscle contraction.^12^, ^15^, ^16^ When investigating motor adaptations to pain, this paradoxical association between movement and pain may lead to a perception of movement as less threatening than what occurs when the movement elicits pain. Thus, it is possible that experimental approaches that induce movement‐evoked pain will result in larger motor adaptations to pain compared to tonic pain.

Painful electrical stimulation has traditionally been used to induce transient pain.^17^, ^18^ More recently, this technique has been used to investigate whether motor adaptations are a purposeful strategy to reduce the noxious stimulation.^19^, ^20^, ^21^ For instance, by modulating in real time the amplitude of the stimulation based on the load applied to the right leg on the ground, we showed that participants were able to decrease their perceived pain intensity by redistributing the amount of weight between legs.^21^ Together with the fact that painful electrical stimulation delivered as low‐frequency sinusoidal waveforms results in minimal habituation over time,^21^ this experimental pain model offers a unique opportunity to directly compare motor adaptation to tonic and movement‐evoked pain.

The purpose of this study was to test the hypothesis that movement‐evoked pain induces larger motor adaptation than tonic pain. Movement‐evoked pain was induced by modulating the intensity of the electrical stimulation in real time according to the amount of knee extension torque. Since the presence of pain is known to reduce maximal torque production,^15^, ^22^ we hypothesized that knee extension torque would be reduced more during movement‐evoked pain compared to tonic pain. Additionally, since a motor adaptation to pain model suggests that the activity of antagonist may be increased by pain,^23^ we sought to determine whether the decrease in knee extension torque production during pain was due to increased activation of the antagonist muscle (biceps femoris).

## 2 METHODS

### 2.1 Participants

Twenty‐one healthy volunteers (12 males and 9 females; age: 25.6 ± 6.9 years; height: 175.5 ± 8.7 cm; mass: 71.1 ± 16.9 kg) were recruited from the staff and student population at University of Birmingham. Based on changes in isometric knee extension maximal voluntary contraction (MVC) from 97.4 ± 1.7% at baseline to 88.1 ± 14.4% during experimentally‐induced pain (Cohen's *d*_z_ = 0.67 for a correlation between groups = 0.3),^15^ an a priori sample size calculation revealed that 20 participants would be needed to obtain a power of 80%. For this analysis, G*Power 3.1.9.7^24^ was used with the test family, statistical test and type of power analysis defined as “*t*‐tests (two tails),” “difference between matched pairs,” and “a priori”, respectively. All participants were free of lower limb injury, had no history of lower limb surgery or disorders and were not taking pain or antidepressants medication. In addition, participants were asked to not consume caffeine prior to the experimental sessions. This study was approved by the Research Ethics Committee at the University of Birmingham (ERN_19‐1018A) and conformed to the latest Declaration of Helsinki. All participants provided written informed consent prior to the experimental procedures and completed a pre‐test health screen to ensure no contraindications to exercise.

### 2.2 Study design

In a repeated measures design, participants took part in two experimental sessions, separated by at least 3 days (median [1st quartile, 3rd quartile]: 9 [7, 16] days). Participants were asked to avoid any strenuous exercise before each session. All experiments were conducted at the School of Sport, Exercise and Rehabilitation Sciences (University of Birmingham) from June 29, 2021 to October 9, 2021. Motor adaptation was quantified as changes in maximal torque production of the knee extensors, which has consistently been shown to decrease in response to tonic experimental pain^15^, ^22^, ^25^ and in individuals with clinical knee pain such as patellofemoral pain.^26^ During both sessions, participants were asked to perform isometric knee extension MVCs without pain (baseline), during experimental knee pain (pain) and after pain (post‐pain). In both sessions, the experimental knee pain was induced using electrical stimulation, but in one session the stimulation intensity was tonic (i.e., constant) and in the other session the stimulation intensity was movement‐evoked (i.e., modulated proportionally to the amount of knee extension torque). To control for order effects, the order of sessions was randomized among participants using the website random.org.

### 2.3 Data collection

**FIGURE 1:**
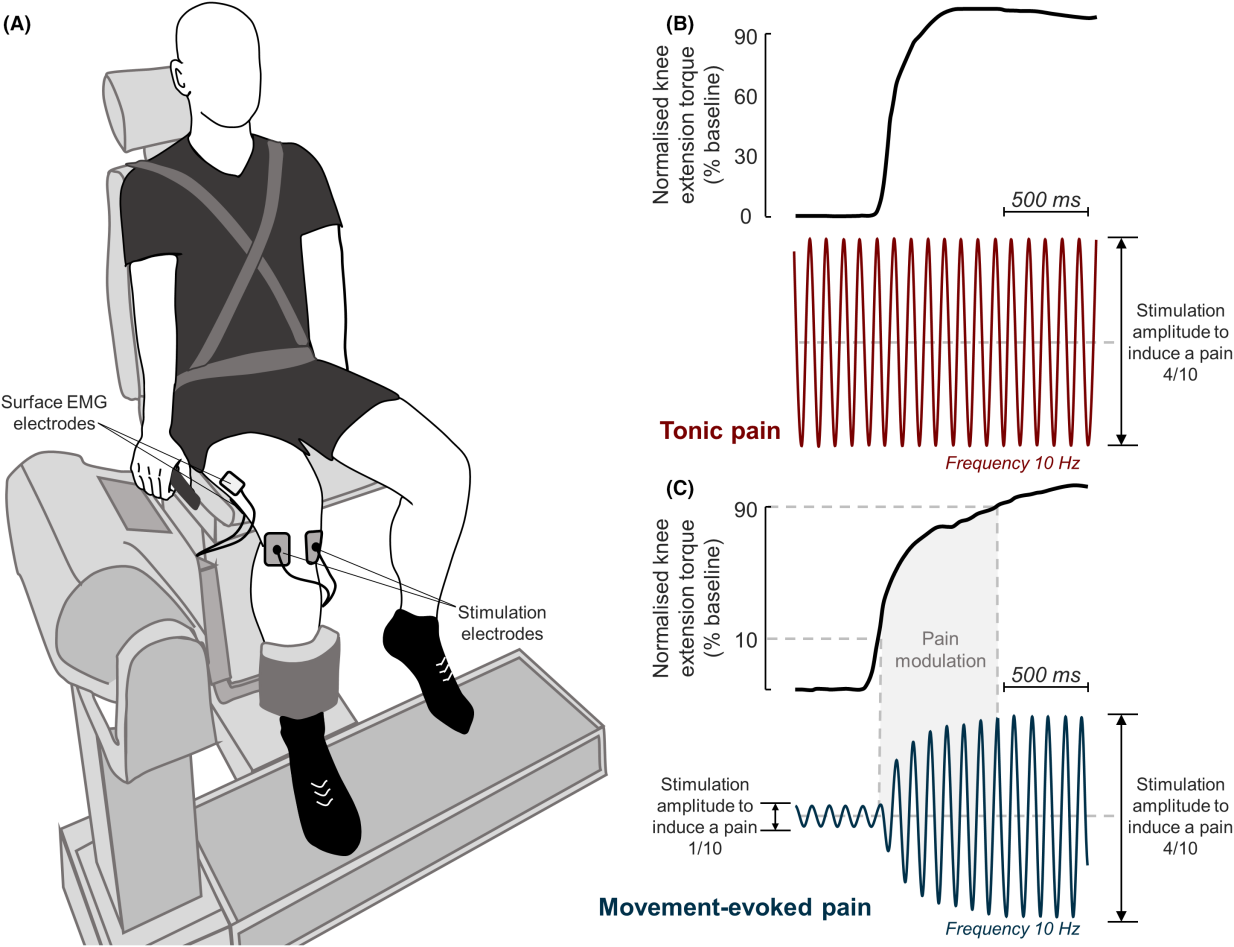
Experimental setup. (A) shows the position of the participants on the dynamometer chair and the position of stimulation and electromyography (EMG) electrodes. (B) illustrates the tonic pain condition in which the intensity of the stimulation painful (10 Hz sine wave) was constant and set to induce a pain intensity of 4/10 (numeric rating scale). (C) illustrates the movement‐evoked pain condition in which the stimulation intensity was modulated proportionally to the knee extension torque exerted by participants. When the knee torque produced was ≤ than 10% and ≥ than 90% of baseline peak torque, the amplitude of stimulation was set to induce a pain intensity of 1/10 and 4/10, respectively. When the torque produced was between 10% and 90% of the baseline peak torque (gray area), the amplitude of painful electrical stimuli was modulated linearly between the intensities required to generate a pain of 1/10 and 4/10.

At the beginning of each session, participants were comfortably seated and secured onto a dynamometer chair (Biodex System 3), with their dominant (19 right leg and 2 left leg; determined by the preferred leg used to kick a ball)^15^; knee flexed at 80° (0° means full extension) and aligned as coaxially as possible to the dynamometer axis of rotation (Figure 1A). Participants were then familiarized with the task by performing three submaximal isometric contractions of knee extension^27^ at approximately 50%, 75%, and 100% of their self‐perceived maximum effort, with a rest‐in‐period of 1 min. After this warm up, participants performed isometric knee extension MVCs for baseline, pain, and post‐pain conditions. Three repetitions were conducted for each condition, with a 2‐min rest between repetitions. All participants were instructed to perform the MVC as hard and as fast as possible and to exert force for 5 s. This amount of time was standardized using a timer, and the investigators verbally encouraged the participant throughout the task. When the 5 s elapsed, the investigators stopped encouraging the participant. Torque signals were sampled in volts at 2000 Hz using a PCI‐6229 board (National Instruments).

Two pairs of bar electrodes (10 × 1 mm electrode size; 10 mm interelectrode distance; Delsys Bagnoli, Delsys Inc.) were used to sample surface EMG signals from the vastus lateralis (VL) and biceps femoris (BF) muscles during the MVCs. We chose to assess the VL muscle as this muscle has the highest physiological cross‐sectional area among the quadriceps and therefore a greater potential to generate force.^28^, ^29^ The electrodes were positioned in accordance with SENIAM guidelines^30^ and fixed to the skin with a bi‐adhesive foam. A reference electrode (16 mm diameter; AMBU WhiteSensors) was placed on the head of the fibula. Prior to electrode placement, the skin over the VL and BF was cleaned with abrasive gel (NUprep, Weaver and Co), and shaved when necessary. Bipolar surface EMG signals were amplified by a factor of 100 and digitized at 2000 Hz using the PCI‐6229 board (16‐bit A/D converter; ± 10 V range). The torque signal provided by the dynamometer and EMG signals were sampled synchronously using a custom written Simulink model (Matlab, The MathWorks Inc.).

### 2.4 Painful electrical stimulation

Knee pain was induced by electrical stimulation delivered through two surface electrodes (3.0 × 3.5 cm, TE0N1S3545, SpesMedica) placed on the medial and lateral aspects of the infrapatellar fat pad,^21^ which was manually identified by palpation (Figure 1A). In line with a previous study,^21^ this location was chosen because the fat pad is highly innervated by nociceptors and electrical stimulation at this location was shown to induce localized pain without muscle twitching. An electrical stimulator designed for constant stimulation (Digitimer DS5 Isolated Bipolar Constant Current Stimulator) was used to deliver sinusoidal waveforms at 10 Hz. At the start of the stimulation, a transition from 0 mA (no stimulation) to the sinusoidal current profile introduces a large artifact on the EMG.^21^ This artifact cannot be removed by high‐pass filtering at 20 Hz because this transition contains frequencies larger than the frequency of the sinusoid used for the electrical stimulation. In order to smooth the transition and reduce the artifact on the EMG, the stimulation signal containing the “no stimulation” and the sinusoidal waveform at 10 Hz was low‐pass filtered at 20 Hz using a sixth order Butterworth filter to remove the high frequencies at the transition. The stimulator was controlled using an analog signal created in a custom written Simulink model and generated at 2000 samples using a PCI‐6229 board with 16‐bit resolution. These stimulation parameters were chosen because minimal habituation of pain intensity ratings over time and minimal stimulation artifacts on the EMG were observed with this configuration in a previous study.^21^ Moreover, delivery of sinusoidal waveforms at 10 Hz, instead of lower frequencies, was considered to provide the most continuous sensation of pain during pilot testing. The stimulation intensity was set to induce a pain intensity of 4/10 measured using a verbal numeric rating scale (NRS) from 0 to 10 (0 being no pain and 10 the worst pain imaginable). We target a pain intensity of 4/10 because is similar to what participants reported in other experimental knee pain studies.^15^, ^31^ This intensity was determined before the baseline condition by an ascending stimulation protocol, which started with a stimulus amplitude of 0.5 mA and increased with steps of 0.5 mA. During this protocol, each painful electrical stimulus was delivered for 2 s with a rest‐in‐period of ~5 s, and the stimulation intensities to induce a pain of 1/10 and 4/10 (NRS) were recorded for each participant.

### 2.5 Experimental task

For the tonic pain condition, the stimulation intensity was constant and set to induce a pain intensity of 4/10 (NRS). Participants were instructed to perform the isometric knee extension MVCs (Figure 1B) approximately 5 s after the start of the stimulation, and the stimulation was stopped approximately 3 s after the end of the MVC. Conversely, for the movement‐evoked pain condition, the stimulation intensity was modulated proportionally to the amount of knee extension isometric torque produced by the participant. Similar to a recently described methodology,^21^ the torque signal was collected and inputted in a Simulink model to modulate the amplitude of the stimulation in near real‐time. The maximal knee extension peak torque obtained at baseline MVCs was used to determine the thresholds to modulate the painful stimulation intensity (Figure 1C). When knee extension torque produced was equal to or lower than 10% of baseline peak torque, the amplitude of stimulation was set to induce a pain intensity of 1/10 NRS (minimal pain). When the torque produced was between 10% and 90% of the baseline peak torque, the amplitude of the painful electrical stimuli was modulated linearly between the intensities required to generate a pain of 1/10 and 4/10 (NRS). Specifically, the 10 Hz sinusoidal waveform of unitary amplitude was scaled proportionally to the instantaneous normalized knee extension torque. Finally, when the torque produced was equal to or higher than 90% of the baseline peak torque, the amplitude of painful electrical stimulation corresponded to a pain intensity of 4/10 (NRS). The average time delay between the input torque and the delivered painful electrical stimuli was 25 ms. Participants were not allowed practice MVCs during pain, that is: the MVCs used for the analyses were the first three each participant performed while experiencing the painful stimulation. After each painful MVC of the knee extensors, participants were asked to rate their perceived peak pain intensity (NRS) before the contraction, as a measure of pain at rest, and during the contraction. As preliminary analyses revealed lower pain ratings during the contraction compared to before the contraction in the tonic pain condition, the last 11 participants were asked to rate their perceived pain also after the contraction to ensure that the decrease in pain during contraction was not due to habituation. No differences were observed in the pain ratings before the contraction across the three MVC trials and, thus, the average of pain ratings across the trials were retained for further analysis.

### 2.6 Data processing

Torque and EMG were analyzed offline using Matlab (The MathWorks Inc.). Raw torque signals were converted to Newton‐meters (Nm) and low‐pass filtered at 10 Hz using a fourth order Butterworth filter. The peak torque was then computed for each MVC as the maximal value identified in the entire torque signal. Visual inspection of all trials for each participant ensured that there were no instances of multiple peaks. In preliminary analyses we observed differences in the duration of contraction performed during painful stimulation, thus we also computed the duration for each contraction and time to peak torque. The duration was calculated as the time elapsed between the onset and offset of the torque production using a threshold of 7.5 Nm.^32^ The time to peak torque was calculated as time elapsed from the torque onset to the peak torque. Bipolar EMG signals were band‐pass filtered with a fourth order Butterworth (20–350 Hz cut‐off frequencies), then the root mean square (RMS) amplitude was calculated to estimate the degree of VL and BF activation during the MVCs. A window of 250 ms starting from the middle of the contraction (2.5 s after the MVC onset) was used to compute RMS values.^15^ We also calculated the RMS amplitude during the rest period before the start of the painful contractions (250‐ms window before the MVC onset). For all variables (peak torque, contraction duration, VL RMS and BF RMS), the average across the three MVCs of each condition (baseline, pain, and post‐pain) was considered for analysis.

### 2.7 Statistical analysis

All statistical analyses were performed in IBM SPSS Statistics (version 29.0). Parametric and non‐parametric analyses were considered for inferential statistics depending on the data normality (Shapiro–Wilk test).

To establish the between‐session reliability (tonic vs. movement‐evoked pain sessions) of the stimulation intensity to induce a pain of 1/10 and 4/10 and of the baseline MVC, we used the intraclass correlation coefficient (ICC) calculated using the two‐way mixed‐effects model and absolute agreement for average measures and interpreted by thresholds (poor: 0.00–0.39; fair: 0.40–0.59; good: 0.60–0.74; excellent: 0.75–1.00). Moreover, regardless of whether the participant started with the tonic pain session or movement‐evoked pain session, we assessed whether a systematic bias existed between days by comparing the baseline MVC and the stimulation intensity to induce a pain of 1/10 and 4/10 between Day 1 and Day 2 (paired *t*‐test or Wilcoxon signed‐rank test).

The Wilcoxon signed‐rank test was used to identify differences in stimulation intensity between conditions, pain ratings before and during the contraction (separately for each condition), and pain ratings during the contraction between tonic and movement‐evoked pain. To ensure that there was no habituation during tonic pain (see Section 2), we also compared the pain ratings before and after the contraction using the Wilcoxon signed‐rank test (*N* = 11).

The main aim of the study was to understand whether changes in muscle force production and activation differed between the movement‐evoked and the tonic pain conditions. To test this, a two‐way repeated measures ANOVA was used to compare main and interaction effects of condition (tonic pain and movement‐evoked pain) and time (baseline, pain, and post‐pain) on peak torque, time to peak torque, contraction duration and muscle activation. Post hoc comparisons within each condition were performed with Bonferroni correction, and interaction effects were decomposed with pairwise contrasts with respect to baseline. For VL RMS and BF RMS, we applied log transformation as the data were not normally distributed. A paired *t*‐test was used to compare the muscle activation during the rest period before the start of the MVCs during pain.

While analyzing the data, we observed that the median pain reported during contraction was significantly lower during tonic pain compared to movement‐evoked pain (see Section 3 below). As this difference could be a confounding factor for our main analysis (difference in torque when exposed to movement‐evoked compared to tonic pain), we performed additional analyses to investigate whether individuals who reported less pain during tonic compared to movement‐evoked pain were those who showed larger differences in knee extension torque between conditions. At the group level, a Spearman correlation was performed to test whether differences in peak torque between conditions were associated with differences in pain ratings between conditions. At the individual participant level, a subgroup analysis was performed to compare the average change in knee extension torque between those individuals who reported a similar pain intensity between conditions (absolute difference lower than 0.5/10; *N* = 6) and those individuals who reported a difference in pain intensity between conditions (absolute difference higher or equal than 0.5/10; *N* = 15). A significant association (group level) or a large difference in knee extension torque between subgroups (individual participant level) would imply that the lower pain reported in the tonic compared to movement‐evoked pain condition was a main factor in the between‐session reduction in knee extension torque production. In addition, we performed Spearman correlations between pain ratings during the contraction and changes in peak torque, separately for each condition. The α threshold for all tests was set at 0.05.

## 3 RESULTS

The analyzed dataset is available as supplementary material (Table S1).

### 3.1 Reliability analysis

We determined the between‐session reliability of torque and stimulation intensity between tonic and movement‐evoked pain sessions. The ICC values (95% confidence interval) for the baseline MVC was 0.97 (0.93, 0.99) and for the stimulation intensities to induce a pain of 1/10 and 4/10 NRS were 0.30 (−0.14, 0.64) and 0.73 (0.43, 0.88), indicating excellent, poor and good reliability between sessions, respectively. In addition, regardless of whether the participant started with the tonic pain session or movement‐evoked pain session, no between‐day systematic bias was observed for baseline MVC measures (t(*20*) = 0.156, *p* = 0.828; Day 1: 211.25 ± 87.60 Nm; Day 2: 212.33 ± 89.82) or stimulation intensities to induce a pain 1/10 (t*(20)* = −0.412, *p* = 0.685; Day 1: 1.52 ± 0.66 mA; Day 2: 1.60 ± 0.66 mA) and 4/10 NRS (Wilcoxon signed‐rank test; *N* = 21; *z* = −0.887, *p* = 0.375; Day 1: 5.67 ± 2.11 mA; Day 2: 6.19 ± 2.81 mA).

### 3.2 Stimulation intensity

No significant difference was observed in the stimulation intensity necessary to induce pain of 4/10 NRS between tonic (median [1st quartile, 3rd quartile]: 5.0 [4.5, 7.0] mA) and movement‐evoked (6.0 [4.5, 7.5] mA) pain conditions (difference: 0.0 [−0.5, 0.5] mA; Wilcoxon signed‐rank test, *N* = 21, *z* = −0.372, *p* = 0.709). When pooling both conditions, the stimulation intensity to induce pain of 4/10 NRS at rest was 5.3 [4.5, 7.5] mA.

### 3.3 Knee pain intensity

**FIGURE 2:**
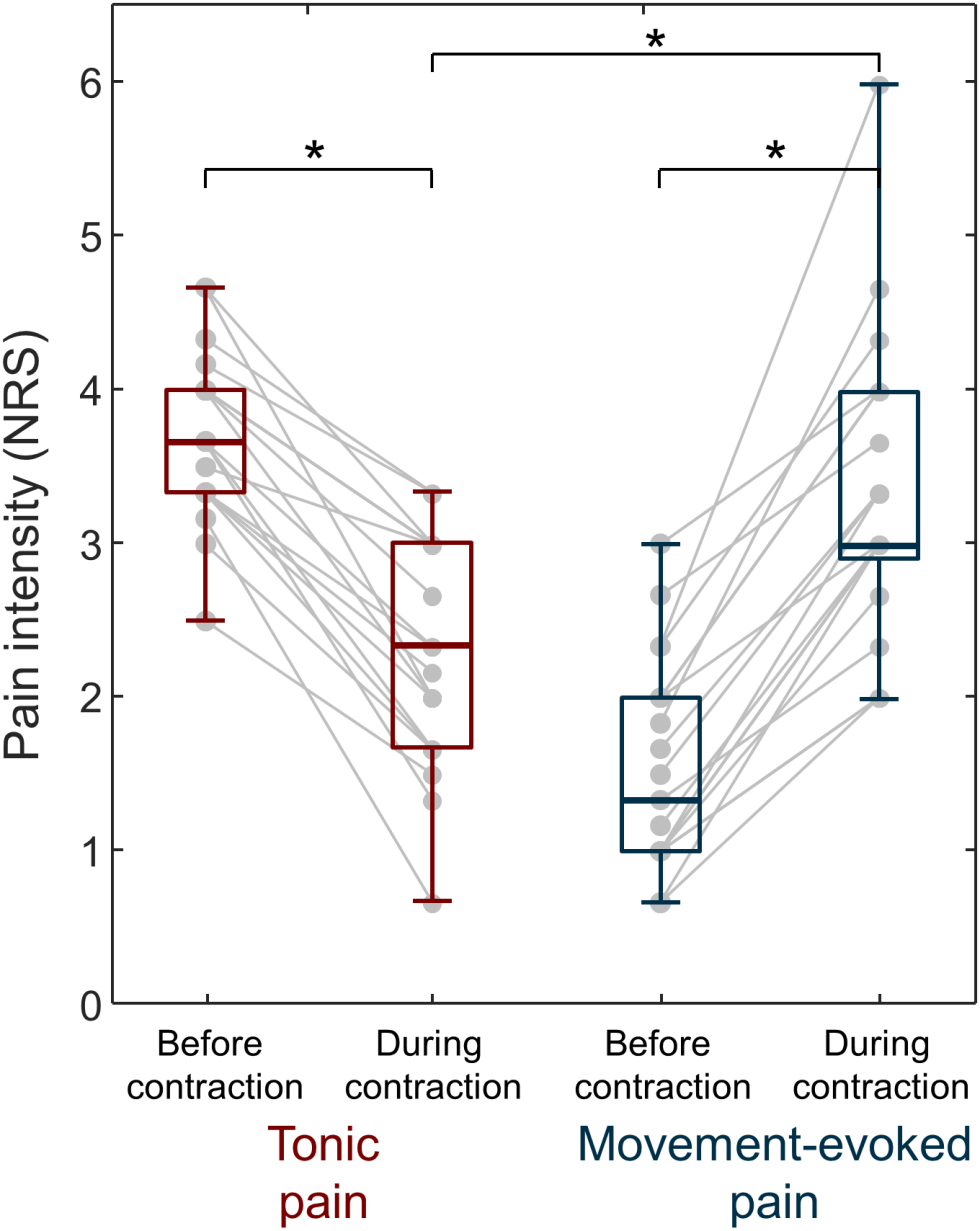
Pain intensity. Pain intensity ratings obtained before and during contractions for tonic pain (red; *N* = 21) and movement‐evoked pain (blue; *N* = 21) conditions. Gray circles identify individual participants. Box and whiskers plots denote median value, interquartile interval, and distribution range. *p < 0.001.

In the tonic pain condition, individuals reported less pain during contraction (2.3 [1.7, 3.0] NRS) than at rest (3.7 [3.3, 4.0] NRS; Wilcoxon signed‐rank test, *N* = 21, *z* = −4.009, *p* < 0.001; Figure 2). Conversely, for the movement‐evoked pain condition, participants reported more pain during contraction (3.0 [3.0, 4.0] NRS) than at rest (1.3 [1.0, 2.0] NRS; Wilcoxon signed‐rank test, *N* = 21, *z* = −4.014, *p* < 0.001; Figure 2) as expected. Perceived pain intensity during contraction was significantly greater for movement‐evoked compared with tonic pain (difference: 1.00 [0.3, 1.7] NRS; Wilcoxon signed‐rank test, *N* = 21, *z* = −3.224, *p* = 0.001; Figure 2). Additionally, in the tonic pain condition, no significant difference was observed in the pain intensity before (3.7 [3.3, 4.0] NRS) and after the contraction (3.7 [2.7, 4.0] NRS; Wilcoxon signed‐rank test, *N* = 11, *z* = −1.472, *p* = 0.141).

### 3.4 MVC peak torque, contraction duration, and muscle activation

The raw values of MVC peak torque, contraction duration and muscle activation measured during baseline, pain, and post are presented in Table 1, separately for each condition.

**TABLE 1:** Average ± standard deviation values of MVC peak torque, muscle activation, and contraction duration.

		MVC peak torque (Nm)	Vastus lateralis activation (μV)	Biceps femoris activation (μV)	Contraction duration (s)	Time to peak torque (s)
Base	Tonic	211.4 ± 84.2	171.7 ± 111.2	23.7 ± 12.6	5.4 ± 0.3	2.1 ± 0.8
	MEP	212.2 ± 93.0	142.49 ± 63.6	20.8 ± 9.2	5.5 ± 0.4	2.4 ± 1.1
Pain	Tonic	185.1 ± 65.5	141.5 ± 87.5	21.1 ± 10.4	5.9 ± 0.4	2.7 ± 0.8
	MEP	174.5 ± 75.0	113.1 ± 53.2	16.9 ± 7.4	5.6 ± 0.4	3.0 ± 1.2
Post	Tonic	206.0 ± 74.9	144.0 ± 70.2	22.8 ± 11.7	5.5 ± 0.3	2.2 ± 0.8
	MEP	206.9 ± 83.2	136.3 ± 62.7	19.4 ± 9.6	5.5 ± 0.4	2.5 ± 1.0

Abbreviation: MEP, movement‐evoked pain.

**FIGURE 3:**
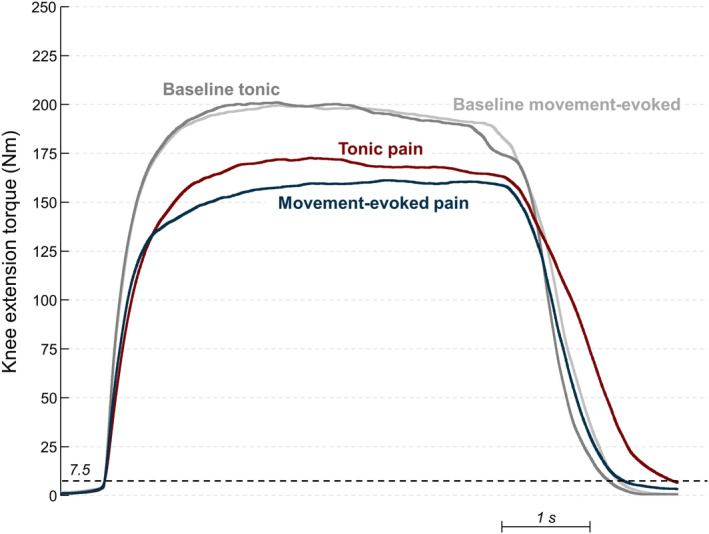
Effect of tonic pain (blue) and movement‐evoked pain (red) on maximal knee extension torque averaged across all participants. Average maximal knee extension torque acquired during baseline are shown separately for tonic (dark gray) and movement‐evoked (light gray) conditions. The black dashed line indicates the torque threshold (7.5 Nm) used to define the onset and offset of torque production.

The effect of tonic and movement‐evoked experimental pain on maximal knee extension torque and contraction duration is displayed in Figure 3. For both tonic and movement‐evoked pain, there was a reduction in the knee extension torque during painful MVCs compared to baseline (Figure 3). A larger reduction was observed when the stimulation intensity was modulated by the knee extension torque (i.e., movement‐evoked pain; dark blue line in Figure 3) compared to tonic pain (dark red line in Figure 3). As shown in Figure 3, the contraction duration was longer for tonic pain compared with the movement‐evoked pain condition.

**FIGURE 4:**
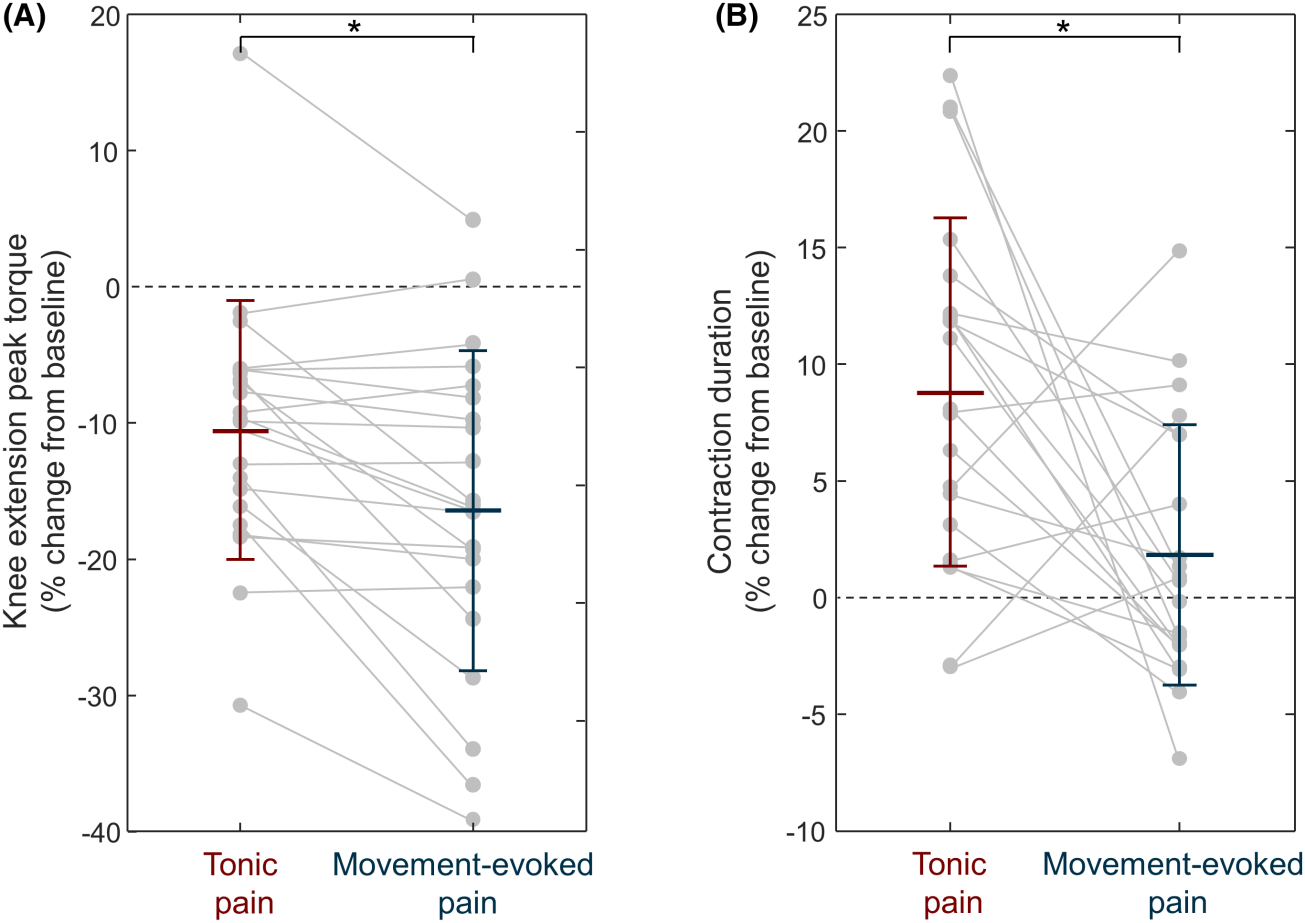
Effect of tonic pain (red; *N* = 21) and movement‐evoked pain (blue; *N* = 21) conditions on (A) knee extension peak torque and (B) contraction duration. The values are the percentage change from baseline. Gray circles identify individual participants. Horizontal line and whiskers denote mean and standard deviation. *p < 0.01.

**FIGURE 5:**
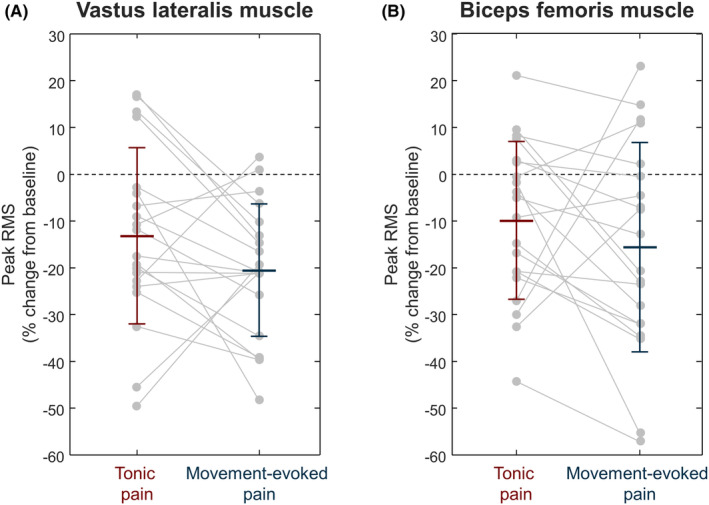
Effect of tonic pain (red; *N* = 20) and movement‐evoked pain (blue; *N* = 20) on peak root mean square (RMS) amplitude of (A) vastus lateralis and (B) biceps femoris muscles. The values are the percentage change from baseline. Gray circles identify individual participants. Horizontal line and whiskers denote mean and standard deviation. *p < 0.05.

These results were confirmed statistically (Figures 4 and 5). Two‐way repeated measures ANOVA identified an interaction effect of painful stimulation and time on the torque produced (*F* (2,40): 5.76, *p* = 0.006). Torque production decreased during pain compared to baseline (tonic: −26.3 ± 26.9 Nm, *p* < 0.001; movement‐evoked pain: −37.7 ± 32.2 Nm, *p* < 0.001), and it returned to baseline in the trials post pain (tonic: −5.4 ± 21.6 Nm, *p* = 0.786; movement‐evoked pain: −5.3 ± 17.8 Nm, *p* = 0.568). The torque reduction from baseline was 5.9 ± 7.5% larger during movement‐evoked than tonic pain (*N* = 21, pairwise contrast with respect to baseline, *p* < 0.001), but no difference was observed between conditions during post pain (0.4 ± 11.4%, *N* = 21, pairwise contrast with respect to baseline, *p* = 0.967). Results were comparable when the highest MVC trial instead of the average of the three MVC trials was considered (4.9 ± 7.5% difference, *N* = 21, pairwise contrast with respect to baseline, *p* < 0.005). Two‐way repeated measures ANOVA also identified a main effect of time on the time to peak torque (*F* (2,40): 16.81, *p* < 0.001), but no main effect of painful stimulation (*F* (1,20): 3.54, *p* = 0.074) or interactions (*F* (2,40): 0.02, *p* = 0.979). Time to peak torque increased during pain compared to baseline (0.6 ± 0.8 s, *p* < 0.001), and it returned to baseline in the post‐pain (0.1 ± 0.7 s, *p* = 0.633). An interaction effect of painful stimulation and time on the contraction time was identified by the two‐way repeated measures ANOVA (*F* (2,40): 6.90, *p* = 0.003). Post hoc tests revealed that contraction duration significantly increased during tonic pain (0.5 ± 0.4 s, *p* < 0.001) and returned to baseline in the trials post pain (0.1 ± 0.3 s, *p* = 1.000). Conversely, no significant changes in contraction duration were observed during movement‐evoked pain compared to baseline (*p* = 0.607) and post pain (*p* = 1.000). The change from baseline in contraction duration differed between tonic and movement‐evoked pain (7.0 ± 10.1% longer for tonic pain, pairwise contrast with respect to baseline, *p* = 0.005).

EMG data from one participant was excluded due to technical issues with the data acquisition. This participant was included in all the other analyses. When considering the muscle activation during the rest period before the start of the painful contractions, there were no differences between tonic and movement‐evoked pain conditions, both for the VL (N = 20, *t*(*19*) = 0.36, *p* = 0.722) and BF (*N* = 20, *t*(*19*) = 0.11, *p* = 0.917) muscles. Two‐way repeated measures ANOVA revealed a main effect of time on the VL activation (*F* (2,38): 17.30, *p* < 0.001), but no effect of painful stimulation (*F* (1,19): 3.71, *p* = 0.069) or interactions (*F* (2,38): 2.03, *p* = 0.146). VL activation decreased during pain compared to baseline (−29.79 ± 36.62 μV, *p* < 0.001), and it returned to baseline in the trials post pain (−16.95 ± 43.26 μV, *p* = 0.093). For the BF activation, there was a main effect of time (*F* (2,38): 8.90, *p* < 0.001) and painful stimulation (*F* (1,19): 8.18, *p* = 0.010), but no interactions (*F* (2,38): 0.75, *p* = 0.479). Post hoc tests revealed that BF activation significantly decreased during pain (−3.25 ± 4.76 μV, *p* = 0.005) and returned to baseline in the trials post pain (−1.22 ± 4.65 μV, *p* = 0.322).

The following analyses were performed to test whether the differences in torque between conditions may be due to the lower pain intensity reported during contraction while exposed to tonic compared to movement‐evoked pain. The between‐condition difference in perceived pain intensity was not associated with the between‐condition difference in normalized knee extension peak torque reduction (Spearman correlation, *ρ* = 0.28, *p* = 0.211), indicating that participants who reported more pain during the movement‐evoked pain did not show a larger decrease in knee extension torque than those who reported similar pain intensity between sessions. This was also confirmed at an individual participant level. The *N* = 6 participants who reported similar pain ratings during tonic and movement‐evoked pain (absolute difference lower than 0.5/10 NRS) showed on average a 6.6% lower torque production during movement‐evoked compared to tonic pain. This difference is similar to the 5.7% difference observed in the *N* = 15 participants who reported lower pain during tonic compared to movement‐evoked pain (absolute difference higher or equal to 0.5/10 NRS). Moreover, there were no significant correlations between reductions in MVC and pain ratings neither for tonic (Spearman correlation, ρ = 0.25; *p* = 0.274) nor for movement‐evoked pain (Spearman correlation, ρ = 0.32; *p* = 0.162) conditions.

## 4 DISCUSSION

We investigated whether pain that increases proportionally to the knee extension torque has a larger effect on maximal knee extension torque than a constant painful stimulation. Both tonic pain and movement‐evoked pain decreased maximal knee extension torque, but larger reductions were observed with movement‐evoked pain. Contraction duration was longer during tonic pain, possibly due to the pain‐relieving effect of muscle contraction. Our findings indicate a larger decrease in force production when force production elicits pain, compared to when it does not. In addition, our findings suggest that the same painful stimulus applied continuously or in association with movement elicits different motor adaptations, both consistent with an attempt to reduce pain. These findings provide mechanistic support for studies investigating whether exercise prescription should differ when pain is associated to movement or not.

### 4.1 Electrical stimulation as a tonic pain model

Our results support the use of electrical painful stimulation to induce tonic pain. First, the reduction in maximal knee extension torque observed during tonic pain (~11%; Figure 4A) was of a similar magnitude as reported previously in studies using injections of hypertonic saline solution to induce experimental knee pain^15^, ^25^ with peak torque reduction of at least 5% observed in 86% of the participants, compared to 50% in other studies.^15^, ^25^ Second, in line with previous findings using injections of hypertonic saline solution,^12^, ^15^ participants reported less pain during contraction than at rest. Because of these similarities, our results support the use of electrical stimulation to induce tonic pain.

### 4.2 Movement‐evoked pain induces larger motor adaptation to pain than tonic pain

Compared to the ~11% reduction in maximal knee extension torque observed during tonic pain, movement evoked pain resulted in a significantly larger reduction of reduction of 17%. Because of the direct association between larger torque production and increase in pain, the larger reduction in knee extension in the movement‐evoked condition is consistent with theories that adapted motor responses occurs as a purposeful strategy to reduce pain.^4^ A possible explanation for the larger decrease in torque could be that, due to its close association with the increase in pain intensity, force production was considered a larger threat for body integrity^33^ in the movement‐evoked compared to the tonic condition. Research on individuals with lived experience of knee pain report that pain elicited with movement is interpreted as a potential sign of tissue damage, prompting avoidance behavior.^34^ In this study, larger protective behavior may have been promoted by an interpretation of movement‐evoked pain as a precursor of tissue damage,^33^ or because movement‐evoked pain is a more attention‐demanding perception of pain^35^ than tonic pain. Overall, our results provide experimental evidence that pain intensity that increases with force production elicits larger protective/avoidance behavior than tonic stimulation, which is consistent with multiple theories of motor adaptation to pain.^4^, ^23^, ^36^

### 4.3 Effect of pain on vastus lateralis (agonist) and biceps femoris (antagonist) muscles

Since a motor adaptation to pain model suggests that the activity of antagonist may be increased during pain,^23^ we investigated whether the reduction in knee extension torque was associated to increased activation of the biceps femoris. Instead, in line with other studies,^15^, ^37^ our results support a reduction in muscle activation of both vastus lateralis (agonist) and biceps femoris (antagonist), suggesting generalized inhibition of both agonist and antagonist muscles. Our results indicate that the lower torque production during movement‐evoked pain was not due to an increased activation of the antagonist muscles and add to the evidence against theories that predict a systematic increase of activation of the antagonist muscle.^23^

### 4.4 Different adaptation to movement‐evoked and tonic pain

Despite a comparable decrease in time to peak torque during tonic or movement‐evoked pain, contraction duration was longer when participants were exposed to tonic pain, likely due to a prolonged submaximal effort after the MVC was completed (Figure 2). It should be noted that we explicitly encouraged participants to perform the MVC for 5 s but did not ask them to stop exerting force at 5 s. The opposite adaptation (contraction for less time) could have been expected during the movement‐evoked pain condition, but this was not possible due to the instructions to maximally contract for at least 5 s; importantly, the increased contraction time was not observed when exposed to movement‐evoked pain. Considering that during tonic pain participants reported lower pain intensity during contraction than at rest, participants may have increased contraction duration in an attempt to reduce pain intensity for longer. It is unclear whether tonic pain relief with contraction is mediated by gating of the painful stimuli by activation of non‐nociceptive afferents,^38^ lower attention to the painful stimulus^35^ or because the participants felt that they “took action”.^19^ Regardless, these results show that participants adapted to tonic pain by increasing contraction duration, and to movement‐evoked pain by decreasing torque production, and both strategies are in line with a purposeful choice to reduce pain. This finding adds to existing literature supporting the specificity of motor adaptation to pain, for instance when the painful stimulus is induced in different tissues around the knee,^31^ or in deep or superficial tissues.^39^ Overall, our results support the notion of variable motor adaptation to pain, including reduced muscle activation and altered contraction duration,^4^ and further highlight how the association between a painful stimulus and movement is a key factor in how individuals adapt to pain.

### 4.5 Differences in pain intensity during movement‐evoked and tonic pain

Despite standardization of the stimulation intensity, participants reported higher pain intensity during movement‐evoked compared to tonic pain during the MVCs. This could be explained by both physiological and cognitive mechanisms. The firing rate of C nociceptors was shown to be larger when heat painful stimuli were applied with shorter rise time.^40^ It is possible that, despite comparable stimulation amplitude, the quick increase in stimulation amplitude during movement‐evoked pain resulted in larger firing rates of the nociceptors, and therefore larger reported pain compared to the tonic pain condition. An alternative and possibly co‐existing explanation could be a role of cognitive mechanisms, and especially attention.^35^ For example, with tonic pain, less attention might be directed to the nociceptive stimulus because it was not associated with the contraction, therefore constant and highly predictable. It could be argued that since higher pain intensities have been shown to result in larger decreases in knee extensor torque,^22^ the larger decrease in torque production during movement‐evoked pain could be due to the higher pain intensity, rather than the association between movement and pain. However, as participants who reported higher pain intensity during movement‐evoked compared to tonic pain showed similar decreases in muscle force to participants who reported comparable pain intensity between sessions, the differences in torque production observed between tonic and movement‐evoked pain are unlikely to be due to differences in pain intensity.

### 4.6 Perspectives

Both tonic and movement‐evoked pain are commonly described in individuals with musculoskeletal pathologies^13^ and after knee surgery.^41^ For instance, patellofemoral pain is diagnosed as pain that is aggravated with activities that load the patellofemoral joint such as single leg squats,^7^ but in some individuals pain is also exacerbated by prolonged sitting postures.^8^ Similarly, pain at rest and during movement have been showed to be different constructs in individuals after surgery such as knee replacement.^41^ While traditional experimental models of tonic pain have successfully replicated some of the motor adaptations observed in individuals with musculoskeletal disorders, our results suggest that motor adaptations to pain may differ or be larger if induced with movement‐evoked pain. At a mechanistic level, our findings of different adaptation to tonic and movement‐evoked support recent work on individuals with patellofemoral pain highlighting the need to report pain experienced during multiple pain‐provoking tasks^42^ and training interventions specifically targeting painful movements.^43^ These findings may contribute to the development of more effective exercise prescription in the long term.

### 4.7 Limitations

It is well known that electrical stimulation lacks spatial selectivity and, thus, it is possible that some of our results are explained by the activation of non‐nociceptive afferents. However, the changes in pain intensity with contraction and the decrease in torque observed are very similar to those observed after hypertonic saline solution injection, which partially support the validity of the model used. Moreover, painful electrical stimulation is to our knowledge the only exogenous pain model that allows to directly compare movement‐evoked and tonic pain using the same experimental model, and any effect on non‐nociceptive afferents is likely to be similar between the two types of stimulation. With respect to the time of contraction, our protocol gave an indication of a minimum time participants had to contract for. This allowed them to contract for longer (as observed in the case of tonic pain), but not to contract for shorter since they were verbally prompted to contract for at least 5 s. Whether people exposed to movement‐evoked pain choose to contract for less time in order to limit pain still needs to be investigated. Although we did not control for menstrual cycle, compelling evidence indicated that the menstrual cycle did not affect the MVC torque production in females.^44^ In this study, participants were asked to recall their pain before, during, and after the MVC after task completion. Pain ratings collected during the task may have been more accurate. However, asking participants to rate their pain intensity during the MVC task may have resulted in non‐maximal efforts, which would have impacted the muscle force production data. Finally, the stimulation intensity required to induce a pain intensity of 1/10 demonstrated poor reliability between days. This could be due to the difficulty to assess stimuli that induce a very mild pain, to differences in pain induced for a certain stimulation amplitude due to slight differences of electrode placement or skin impedance, or to the small variance of the data compared to the stimulation intensity to induce a pain of 4/10.

## 5 CONCLUSIONS

Our results indicate that a direct association between increased muscle force production and increased pain intensity was a more potent stimulus to reduce knee extension torque than tonic pain. The larger reduction in knee extension torque was not due to increased neural drive to the antagonist (biceps femoris) muscle. Considering the longer contraction time observed during tonic pain, our results suggest that tonic and movement‐evoked pain induce different motor adaptation, both consistent with a purposeful strategy to limit pain.

## FUNDING INFORMATION

This study was funded by a Wellcome Trust Institutional Strategic Support Fund (AG).

## CONFLICT OF INTEREST STATEMENT

The authors declare no conflicts of interest.

## PATIENT CONSENT STATEMENT

All participants provided written informed consent prior to the experimental procedures.

## Supporting information


Table S1.

## Data Availability

The data that supports the findings of this study are available in the supplementary material of this article.

## References

[1] Kettunen JA , Kvist M , Alanen E , Kujala UM . Long‐term prognosis for Jumper's knee in male athletes a prospective follow‐up study. Am J Sports Med. 2002;30:689‐692. 12239003 10.1177/03635465020300051001

[2] Docking S , Rio E , Cook J , Orchard J , Fortington L . The prevalence of Achilles and patellar tendon injuries in Australian football players beyond a time‐loss definition. Scand J Med Sci Sports. 2018;28:2016‐2022. 29572969 10.1111/sms.13086

[3] Brinlee AW , Dickenson SB , Hunter‐Giordano A , Snyder‐Mackler L . ACL reconstruction rehabilitation: clinical data, biologic healing, and criterion‐based milestones to inform a return‐to‐sport guideline. Sports Health. 2022;14:770‐779. 34903114 10.1177/19417381211056873 PMC9460090

[4] Hodges PW , Tucker K . Moving differently in pain: a new theory to explain the adaptation to pain. Pain. 2011;152:S90‐S98. 21087823 10.1016/j.pain.2010.10.020

[5] Corbett DB , Simon CB , Manini TM , George SZ , Riley JL , Fillingim RB . Movement‐evoked pain: transforming the way we understand and measure pain. Pain. 2019;160:757‐761. 30371555 10.1097/j.pain.0000000000001431 PMC6424644

[6] Fullwood D , Means S , Merriwether EN , Chimenti RL , Ahluwalia S , Booker SQ . Toward understanding movement‐evoked pain (MEP) and its measurement: a scoping review. Clin J Pain. 2021;37:61‐78. 33093342 10.1097/AJP.0000000000000891 PMC7708514

[7] Crossley KM , Stefanik JJ , Selfe J , et al. 2016 patellofemoral pain consensus statement from the 4th international patellofemoral pain research retreat, Manchester. Part 1: terminology, definitions, clinical examination, natural history, patellofemoral osteoarthritis and patient‐reported outcome m. Br J Sports Med. 2016;50:839‐843. 27343241 10.1136/bjsports-2016-096384 PMC4975817

[8] Collins NJ , Vicenzino B , van der Heijden RA , van Middelkoop M . Pain during prolonged sitting is a common problem in persons with patellofemoral pain. J Orthop Sports Phys Ther. 2016;46:658‐663. 27374012 10.2519/jospt.2016.6470

[9] Scattone Silva R , Purdam CR , Fearon AM , et al. Effects of altering trunk position during landings on patellar tendon force and pain. Med Sci Sports Exerc. 2017;49:2517‐2527. 28704344 10.1249/MSS.0000000000001369

[10] Rio E , Kidgell D , Purdam C , et al. Isometric exercise induces analgesia and reduces inhibition in patellar tendinopathy. Br J Sports Med. 2015;49:1277‐1283. 25979840 10.1136/bjsports-2014-094386

[11] Liew BXW , Del VA , Falla D . The influence of musculoskeletal pain disorders on muscle synergies—a systematic review. PLoS One. 2018;13:1‐20. 10.1371/journal.pone.0206885 PMC6218076 30395599

[12] Ford B , Halaki M , Diong J , Ginn KA . Acute experimentally‐induced pain replicates the distribution but not the quality or behaviour of clinical appendicular musculoskeletal pain. A systematic review. Scand J Pain. 2021;21:217‐237. 34387953 10.1515/sjpain-2020-0076

[13] Perrot S , Cohen M , Barke A , Korwisi B , Rief W , Treede RD . The IASP classification of chronic pain for ICD‐11: chronic secondary musculoskeletal pain. Pain. 2019;160:77‐82. 30586074 10.1097/j.pain.0000000000001389

[14] Cardoso TB , Pizzari T , Kinsella R , Hope D , Cook JL . Current trends in tendinopathy management. Best Pract Res Clin Rheumatol. 2019;33:122‐140. 31431267 10.1016/j.berh.2019.02.001

[15] Salomoni S , Tucker K , Hug F , McPhee M , Hodges P . Reduced maximal force during acute anterior knee pain is associated with deficits in voluntary muscle activation. PLoS One. 2016;11:1‐14. 10.1371/journal.pone.0161487 PMC4999173 27559737

[16] Ford B , Cohen M , Halaki M , Diong J , Ginn KA . Experimental shoulder pain models do not validly replicate the clinical experience of shoulder pain. Scand. J Pain. 2019;20:167‐174. 10.1515/sjpain-2019-0055 31444968

[17] Tucker K , Larsson AK , Oknelid S , Hodges P . Similar alteration of motor unit recruitment strategies during the anticipation and experience of pain. Pain. 2012;153:636‐643. 22209423 10.1016/j.pain.2011.11.024

[18] Hodges PW , Mellor R , Crossley K , Bennell K . Pain induced by injection of hypertonic saline into the infrapatellar fat pad and effect on coordination of the quadriceps muscles. Arthritis Care Res (Hoboken). 2009;61:70‐77. 10.1002/art.24089 19116977

[19] Bergin M , Tucker K , Vicenzino B , Hodges PW . ‘Taking action’ to reduce pain‐has interpretation of the motor adaptation to pain been too simplistic? PLoS One. 2021;16:1‐19. 10.1371/journal.pone.0260715 PMC8654166 34879091

[20] Bertrand‐Charette M , Jeffrey‐Gauthier R , Roy JS , Bouyer LJ . Gait adaptation to a phase‐specific nociceptive electrical stimulation applied at the ankle: a model to study musculoskeletal‐like pain. Front Hum Neurosci. 2021;15:1‐11. 10.3389/fnhum.2021.762450 PMC8718644 34975433

[21] Gallina A , Abboud J , Blouin JS . A task‐relevant experimental model to target motor adaptation to pain. J Physiol. 2021;599:2401‐2417. 33638152 10.1113/JP281145

[22] Henriksen M , Rosager S , Aaboe J , Graven‐Nielsen T , Bliddal H . Experimental knee pain reduces muscle strength. J Pain. 2011;12:460‐467. 21146464 10.1016/j.jpain.2010.10.004

[23] Lund JP , Donga R , Widmer CG , Stohler CS . The pain‐adaptation model a discussion of the relationship between chronic musculoskeletal pain and motor activity. Can J Physiol Pharmacol. 1991;69:12‐694. 10.1139/y91-102 1863921

[24] Faul F , Erdfelder E , Lang AG , Buchner A . G*power 3: a flexible statistical power analysis program for the social, behavioral, and biomedical sciences. Behav Res Methods. 2007;39:175‐191. 17695343 10.3758/bf03193146

[25] Rice DA , Mannion J , Lewis GN , McNair PJ , Fort L . Experimental knee pain impairs joint torque and rate of force development in isometric and isokinetic muscle activation. Eur J Appl Physiol. 2019;119:2065‐2073. 31332518 10.1007/s00421-019-04195-6

[26] Lankhorst NE , Bierma‐Zeinstra SMA , van Middelkoop M . Factors associated with patellofemoral pain syndrome: a systematic review. Br J Sports Med. 2013;47:193‐206. 22815424 10.1136/bjsports-2011-090369

[27] Hibbert JE , Kulas AS , Rider PM , Domire ZJ . Practice day may be unnecessary prior to testing knee extensor strength in young healthy adults. Int Biomech. 2020;7:58‐65. 33998382 10.1080/23335432.2020.1766997 PMC8130721

[28] Farahmand F , Senavongse W , Amis AA . Quantitative study of the quadriceps muscles and trochlear groove geometry related to instability of the patellofemoral joint. J Orthop Res. 1998;16:136‐143. 9565086 10.1002/jor.1100160123

[29] Lieber RL , Fridén J . Functional and clinical significance of skeletal muscle architecture. Muscle Nerve. 2000;23:1647‐1666. 11054744 10.1002/1097-4598(200011)23:11<1647::aid-mus1>3.0.co;2-m

[30] Hermens HJ , Freriks B , Disselhorst‐Klug C , Rau G . Development of recommendations for SEMG sensors and sensor placement procedures. J Electromyogr Kinesiol. 2000;10:361‐374. 11018445 10.1016/s1050-6411(00)00027-4

[31] Gallina A , Salomoni SE , Hall LM , Tucker K , Jayne Garland S , Hodges PW . Location‐specific responses to nociceptive input support the purposeful nature of motor adaptation to pain. Pain. 2018;159:2192‐2200. 29939960 10.1097/j.pain.0000000000001317

[32] Aagaard P , Simonsen EB , Andersen JL , Magnusson P , Dyhre‐Poulsen P . Increased rate of force development and neural drive of human skeletal muscle following resistance training. J Appl Physiol. 2002;93:1318‐1326. 12235031 10.1152/japplphysiol.00283.2002

[33] Crombez G , Eccleston C , Van Damme S , Vlaeyen JWS , Karoly P . Fear‐avoidance model of chronic pain. Clin J Pain. 2012;28:475‐483. 22673479 10.1097/AJP.0b013e3182385392

[34] Smith BE , Moffatt F , Hendrick P , et al. The experience of living with patellofemoral pain–loss, confusion and fear‐avoidance: a UK qualitative study. BMJ Open. 2018;8:1‐9. 10.1136/bmjopen-2017-018624 PMC5786111 29362256

[35] Eccleston C , Crombez G . Pain demands attention: a cognitive‐affective model of the interruptive function of pain. Psychol Bull. 1999;125:356‐366. 10349356 10.1037/0033-2909.125.3.356

[36] Vlaeyen JWS , Kole‐Snijders AMJ , Boeren RGB , van Eek H . Fear of movement/(re)injury in chronic low back pain and its relation to behavioral performance. Pain. 1995;62:363‐372. 8657437 10.1016/0304-3959(94)00279-N

[37] Bank PJM , Peper CE , Marinus J , Beek PJ , Van Hilten JJ . Motor consequences of experimentally induced limb pain: a systematic review. European J Pain (United Kingdom). 2013;17:145‐157. 10.1002/j.1532-2149.2012.00186.x 22718534

[38] Melzack R , Wall PD . Pain mechanisms: a new theory. Science. 1979;1965(150):971‐979. 10.1126/science.150.3699.971 5320816

[39] Birznieks I , Burton AR , Macefield VG . The effects of experimental muscle and skin pain on the static stretch sensitivity of human muscle spindles in relaxed leg muscles. J Physiol. 2008;586:2713‐2723. 18403422 10.1113/jphysiol.2008.151746 PMC2536575

[40] Yarnitsky D , Simone DA , Dotson RM , Cline MA , Ochoa JL . Single C nociceptor responses and psychophysical parameters of evoked pain: effect of rate of rise of heat stimuli in humans. J Physiol. 1992;450:581‐592. 1432719 10.1113/jphysiol.1992.sp019144 PMC1176139

[41] Srikandarajah S , Gilron I . Systematic review of movement‐evoked pain versus pain at rest in postsurgical clinical trials and meta‐analyses: a fundamental distinction requiring standardized measurement. Pain. 2011;152:1734‐1739. 21402445 10.1016/j.pain.2011.02.008

[42] Glaviano NR , Bazett‐Jones DM , Boling MC . Pain severity during functional activities in individuals with patellofemoral pain: a systematic review with meta‐analysis. J Sci Med Sport. 2022;25:399‐406. 35190263 10.1016/j.jsams.2022.01.004

[43] Salsich GB , Yemm B , Steger‐May K , Lang CE , van Dillen LR . A feasibility study of a novel, task‐specific movement training intervention for women with patellofemoral pain. Clin Rehabil. 2018;32:179‐190. 28750548 10.1177/0269215517723055 PMC5748381

[44] Ansdell P , Brownstein CG , Škarabot J , et al. Menstrual cycle‐associated modulations in neuromuscular function and fatigability of the knee extensors in eumenorrheic women. J Appl Physiol. 2019;126:1701‐1712. 30844334 10.1152/japplphysiol.01041.2018

